# Physiologically Based Pharmacokinetic Modeling to Predict Human Pharmacokinetics of a Novel Mithramycin Analog for Ewing Sarcoma

**DOI:** 10.21203/rs.3.rs-9488905/v1

**Published:** 2026-04-23

**Authors:** Kumar Kulldeep Niloy, Jamie Horn, Nazmul Hasan Bhuiyan, Suhas S. Bhosale, Khaled A. Shaaban, Thomas E. Prisinzano, Jon S. Thorson, Jurgen Rohr, Markos Leggas

**Affiliations:** 1 Department of Pharmacy and Pharmaceutical Sciences, St. Jude Children s Research Hospital, Memphis, Tennessee, United States; 2 Department of Pharmaceutical Sciences, College of Pharmacy, The University of Tennessee Health Science Center, Memphis, Tennessee, United States; 3 Department of Pharmaceutical Sciences, College of Pharmacy, University of Kentucky, Lexington, Kentucky, United States; 4 Center for Pharmaceutical Research and Innovation, College of Pharmacy, University of Kentucky, Lexington, Kentucky, United States

**Keywords:** Physiologically based pharmacokinetic model, First-in-human dose prediction, Interspecies scaling, Mithramycin analog, Ewing sarcoma

## Abstract

**Purpose:**

To develop and verify a physiologically based pharmacokinetic (PBPK) modeling strategy for mithramycin (MTM) and its analog, MTMSA-Trp, with the aim of projecting first-in-human plasma pharmacokinetics and supporting the translational development of MTMSA-Trp for Ewing sarcoma treatment.

**Methods:**

PBPK models were created in GastroPlus^®^ using a middle-out approach, incorporating preclinical pharmacokinetic data from mice, rats, and cynomolgus monkeys. Human clearance was estimated through three methods: an additional clearance approach, allometric scaling, and single-species scaling from monkeys. The model was evaluated using clinical MTM plasma PK data and then employed to project human MTMSA-Trp plasma PK, with tissue predictions considered exploratory.

**Results:**

The additional clearance approach provided the most accurate prediction of human MTM plasma PK. Across all clearance prediction methods, MTMSA-Trp was predicted to achieve 8- to 15-fold higher human plasma exposure than MTM at the same dose. Model-derived liver exposures were 2- to 4-fold higher, with a lower predicted liver partition for MTMSA-Trp; however, these tissue predictions remained sensitive to distribution assumptions. Parameter sensitivity analysis identified the blood-to-plasma ratio as the most influential parameter among those examined.

**Conclusion:**

PBPK modeling supports the projection that MTMSA-Trp will achieve substantially higher plasma exposure than MTM in humans. This empirically developed workflow may inform translational efforts for the first-in-human development of MTMSA-Trp.

## 1. Introduction

MTMSA-Trp, a tryptophan-conjugated analog of mithramycin (MTM), was recently developed to address the poor pharmacokinetics (PK) and dose-limiting toxicities that have prevented MTM from being used as a therapy for Ewing sarcoma (ES) [1–3]. In addition to improved antitumor efficacy, MTMSA-Trp demonstrated substantially lower systemic clearance than MTM across multiple preclinical species [3]. This lower systemic clearance has been attributed to increased lipophilicity and higher plasma protein binding [3]. Together, these properties produced higher plasma exposure than MTM at equivalent doses in rodents and non-human primates, supporting continued translational development of MTMSA-Trp for ES [3].

Building on this preclinical PK characterization, a series of pharmacometric studies has been conducted to support the translational development of MTMSA-Trp [4,5]. A PK model for MTMSA-Trp in athymic nude mice was previously developed using nonlinear mixed-effects modeling to characterize dose-dependent disposition across the therapeutic dose range [4]. That model was subsequently incorporated into a translational tumor growth inhibition/time-to-event (TGI-TTE) framework linking MTMSA-Trp exposure, tumor growth dynamics, and survival in ES xenograft models, in which higher regimen-specific average exposure was associated with longer survival [5].

While these studies highlight the translational promise of MTMSA-Trp, advancing the compound toward first-in-human evaluation requires a robust projection of human plasma PK. PBPK modeling offers a useful framework for this purpose and is widely applied to first-in-human PK translation [6–8]. By integrating drug-specific physicochemical inputs with physiological system parameters, PBPK models can simulate systemic and tissue concentration-time profiles across preclinical species and extrapolate them to humans [9,10]. However, this application also poses challenges for standard tissue-distribution methods because MTM and MTMSA-Trp are unusually large molecules (approximately 1000 to 1500 Da), are highly protein-bound, and exhibit permeability-limited distribution [9,10].

Accordingly, the objective of this study was to develop and verify a middle-out PBPK strategy for MTM and MTMSA-Trp that could support translational human PK projection in the absence of fully defined elimination pathways. Specifically, we sought to establish an empirically calibrated framework for cross-species clearance scaling, verify the workflow against clinical MTM plasma PK extracted from a recent Phase I/II trial [11], and project the first-in-human plasma PK profile of MTMSA-Trp. Because the model was optimized primarily against plasma PK data, tissue exposure predictions, including liver partitioning, were treated as exploratory outputs intended to inform translational planning rather than to establish a definitive clinical therapeutic window.

## 2. Methods

### 2.1. Pharmacokinetic data

Preclinical PK data of MTM and MTMSA-Trp were obtained from previously published PK studies [3]. Briefly, PK studies were conducted in mice at 2 mg/kg by bolus IV injection and in rats at 0.5 mg/kg by bolus IV injection, with cynomolgus monkeys given a 10-minute IV infusion at 6.94 and 13.18 μg/kg for MTM and at 1.34 and 4.19 μg/kg for MTMSA-Trp. PK samples were collected using serial sampling and analyzed using LC-MS/MS. Preclinical PK data were used to develop and verify the PBPK model. Clinical PK data for MTM were extracted from the literature [11] using WebPlotDigitizer v4 (https://automeris.io/) and used to verify the human PBPK model of MTM. A human PBPK model of MTMSA-Trp was developed using the same modeling approach to project human plasma PK and to generate exploratory tissue exposure profiles for comparison with MTM at an equal dose. This dose was tolerated by patients in the clinical trial [11].

### 2.2. Preclinical PBPK model development

PBPK models were developed in GastroPlus^®^ version 9.9 (SimulationPlus). Drug-specific physicochemical parameters (molecular weight, logP, solubility, and pKa) were obtained from ADMET Predictor version 11 (SimulationPlus) or measured experimentally. The species-specific fraction unbound in plasma (fup) was previously measured by equilibrium dialysis [3]. The fup of MTMSA-Trp in mouse and human plasma was below the limit of quantification (<1%) and was set to 0.1% during model development. Blood-to-plasma ratios (BPR) were obtained from ADMET Predictor, assumed based on available data, or adjusted when required to improve model fit. A permeability-limited tissue distribution model was used, and tissue-to-plasma partition coefficients (Kp) were predicted using the Poulin and Theil extracellular method [12]. The primary clearance term in the model was represented as renal clearance (CL_R_). This construct served as an empirical approach in the absence of fully defined elimination pathways. A middle-out modeling strategy was applied for preclinical PBPK model development. The specific permeability-surface area product (SpecPStc) was optimized using mouse plasma PK data and then held constant across species. Observed systemic clearance in each preclinical species was estimated by non-compartmental analysis (NCA) of the observed PK data using PKanalix version 2024R1 (SimulationPlus) and used as an input for CL_R_. Species-specific physiological parameters were obtained from the GastroPlus^®^ built-in databases.

### 2.3. Human PBPK model development

The human PBPK model retained all drug-specific physicochemical parameters and used human-specific fup and BPR. The SpecPStc and Kp prediction methods were carried forward from the preclinical models. Human physiological parameters for a 13-year-old, 50-kg male were selected from the GastroPlus^®^ built-in database to approximate the pediatric/adolescent Ewing sarcoma setting represented by the available clinical MTM comparator data [11]. Three methods were used to project human clearance.

#### Additional clearance approach (Method 1).

In each preclinical species, the observed systemic clearance (CL_Obs_) estimated by NCA was divided into two components: renal clearance (CL_R_) and an additional clearance (CL_add_) as follows:

$$\text{C}{\text{L}}_{\text{obs}}=\text{C}{\text{L}}_{\text{R}}\text{+C}{\text{L}}_{\text{add}}$$

where CL_R_ = fup × GFR i.e. renal filtration-based clearance. To calculate CL_R_, species-specific fup and GFR values were used for each preclinical species. The percentage of CL_Obs_ accounted for by CL_add_ (%CL_add_) was then calculated for mouse, rat, and monkey separately as follows:

$$\text{\%C}{\text{L}}_{\text{add}}\text{=}\left(\text{C}{\text{L}}_{\text{add}}\text{/C}{\text{L}}_{\text{Obs}}\right)\times 100.$$

For human systemic clearance (CL_human_) prediction, renal clearance (CL_R,human_) was predicted similarly (CL_R,human_ = fup_human_ × GFR_human_) where fup,_human_ is the human unbound fraction in plasma, and GFR_human_ is the human GFR for a 13-year-old, 50 kg male, as provided by the GastroPlus^®^ built-in pediatric physiology database.

The additional clearance component in humans was estimated from the geometric average of %CL_add_ across mouse, rat, and monkey. Human clearance (CL_human_) was therefore predicted as follows:

$$\text{C}{\text{L}}_{\text{human}}\text{=}\left[\text{C}{\text{L}}_{\text{R,human}}\text{/}\left(100-\text{Avg.\%C}{\text{L}}_{\text{add}}\right)\right]\times \text{100}.$$

#### Allometric scaling (Method 2).

Observed systemic clearance estimated by NCA in mouse, rat, and cynomolgus monkey was plotted against body weight on a log-log scale and fitted to a simple allometric power function as follows:

$$\text{Clearance}=\text{a}\times \text{B}{\text{W}}^{\text{b}}$$

where BW is the body weight (kg), a is the allometric coefficient, and b is the allometric exponent estimated from the log-log regression fit. The fitted equation was then extrapolated to a human body weight of 50 kg to predict human clearance.

#### Single species scaling from monkey (Method 3).

Human clearance was predicted by scaling the observed clearance of cynomolgus monkey to human using a fixed allometric scaling exponent of 0.75 as follows:

$$\text{C}{\text{L}}_{\text{human}}=\text{C}{\text{L}}_{\text{monkey}}\text{×}{\left(\text{B}{\text{W}}_{\text{human}}\text{/B}{\text{W}}_{\text{monkey}}\right)}^{\text{0.75}}$$

where BW was assumed to be 5 kg for monkey and 50 kg for human. The allometric scaling exponent of 0.75 is commonly reported in the literature [13,14].

Using all three clearance prediction methods, human MTM plasma profiles were simulated and compared with extracted MTM clinical plasma PK data. The clearance prediction method that best described the MTM clinical plasma PK data was retained as the primary human comparator. All three clearance prediction methods were also used to generate human plasma and exploratory liver concentration-time profiles of MTMSA-Trp for 24 hours at 30-minute intervals, and these outputs were compared with the best-fitting human MTM simulation.

### 2.4. Model evaluation

Predictive performance was evaluated by comparing the fold error between predicted and observed area-under-the-curve (AUC) values and plasma concentrations [15,16]. The AUCs up to the last time-point were estimated by NCA using the log-linear trapezoidal rule. The average fold error (AFE) and the root mean square error (RMSE) of log-transformed concentrations were calculated as follows:

$$\text{Fold error}=\frac{\text{Predicted}}{\text{Observed}}\;\text{AFE}=10^{\frac{\sum \log\;\text{fold error}}{\text{n}}}\;\text{RMSE}=\sqrt{\frac{\text{Σ}{\left(\log\;\text{Predicted}-\log\;\text{Observed}\right)}^{2}}{\text{n}}}$$

Where n is the number of time-matched samples. Predictions were considered acceptable if the fold error was within 0.5–2.0 and the visual inspection of predicted versus observed concentration-time profiles was in good agreement.

### 2.5. Parameter sensitivity analysis

Parameter sensitivity analysis (PSA) was conducted on the human PBPK model of MTMSA-Trp to explore the influence of parameters obtained in silico or measured experimentally (BPR, fup, and logP) on predicted human exposure. Each parameter was varied individually over a physiologically plausible range while all other parameters were held constant. Lower and upper BPR values represented the adjusted BPR value for monkey and a generally accepted upper-range assumption for BPR [17]. A 10-fold and 3-fold difference from the input values were chosen for fup and logP, respectively. Simulations were conducted at a 13 μg/kg IV bolus dose in a 13-year-old, 50 kg male using human clearance predicted by allometric scaling (Method 2). This analysis was intended as an illustrative assessment of relative parameter influence rather than formal qualification of the preferred final human model.

## 3. Results

### 3.1. PBPK input parameters

The physicochemical and species-specific input parameters used for PBPK model development are summarized in Table 1. MTMSA-Trp is more lipophilic and exhibits substantially higher plasma protein binding than MTM. Mouse and human BPR values estimated in silico were comparable for both compounds and were lower than those of rats. For MTMSA-Trp, the chemical conjugation (i.e. Trp) also shifted the measured pKa toward physiological pH. The optimized mouse SpecPStc was smaller for MTMSA-Trp, consistent with the lower membrane permeability expected for a larger, more protein-bound molecule.

### 3.2. Preclinical PBPK model development

**Figure 1:**
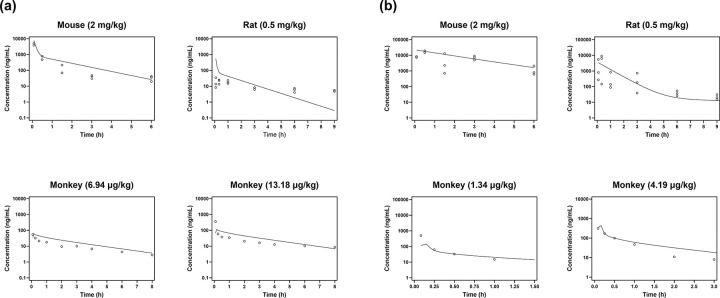
PBPK simulation of plasma PK for (a) MTM and (b) MTMSA-Trp in preclinical species. Mouse served as the calibration species for SpecPStc optimization. Symbols represent observed concentration-time points and solid lines represent PBPK model predictions.

The PBPK models were calibrated in mice and then evaluated against observed plasma concentration-time profiles in rats and cynomolgus monkeys (Fig. 1a and 1b, Table 2). Across mouse, rat, and higher-dose monkey studies, AUC ratios ranged from 1.02 to 1.69, and AFE values ranged from 0.73 to 1.84, generally within the 2-fold acceptance criterion. For cynomolgus monkeys, the BPR of MTM was assumed to be the same as in humans and provided acceptable fits and AUC ratios at both dose levels (Fig. 1a, Table 2). In contrast, initial monkey PBPK simulations for MTMSA-Trp using the in silico human BPR substantially underpredicted the observed monkey plasma concentration-time profiles at both doses (Fig. S1). Sensitivity analysis of monkey BPR for MTMSA-Trp (Fig. S1) indicated that a markedly lower BPR of 0.02 was required to describe the observed monkey PK data, suggesting predominant partitioning into the plasma fraction in cynomolgus monkey blood. After this adjustment, the higher-dose monkey MTMSA-Trp profile met the 2-fold AUC criterion (Table 2), whereas the lower-dose profile remained outside that criterion (AUC ratio 0.46), indicating residual uncertainty at that exposure level.

### 3.3. Human PBPK model verification for MTM

**Figure 2:**
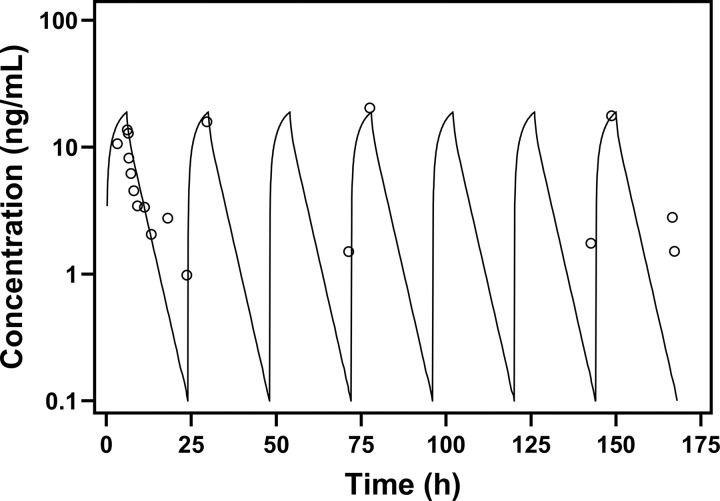
Human plasma PK verification of MTM using human clearance calculated by the additional clearance approach. The solid line represents the PBPK model prediction, and circles represent concentration-time points extracted from reported clinical trial data in which 13 μg/kg MTM was administered as a 6-hour infusion daily for 7 days to a 13-year-old patient with Ewing sarcoma. The PBPK model was simulated using 13-year-old, 50 kg male physiology.

The human MTM PBPK model was evaluated against plasma PK data extracted from ES patients who received 13 μg/kg MTM as a 6-hour IV infusion [11]. Among the three human clearance prediction methods evaluated, the additional clearance approach provided the most accurate description of human MTM plasma PK (Table S1). The predicted AUC was close to the observed AUC, yielding an AUC ratio of 1.05 and an AFE of 0.52. Visual inspection also confirmed close agreement between the predicted and observed plasma concentration-time profiles (Fig. 2). Allometric scaling and single-species scaling from monkey provided poor human plasma PK predictions (Fig. S2, Table S1), and the allometric scaling plot for MTM showed a poor linear regression fit (Fig. S3). Taken together, these findings supported the additional clearance approach, although empirical, as the preferred human clearance prediction method for MTM and justified its use as the primary reference for human MTMSA-Trp plasma projections.

### 3.4. Human plasma PK projection for MTMSA-Trp

**Figure 3:**
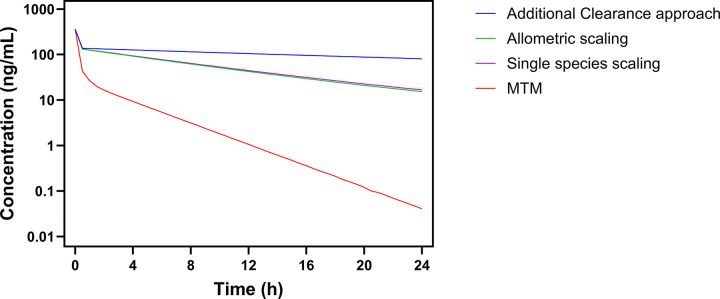
Human plasma PK projection of MTMSA-Trp compared with MTM by PBPK modeling. MTMSA-Trp plasma PK was projected using three human clearance prediction methods and compared with the MTM plasma simulation generated with the additional clearance approach. PBPK simulations were conducted at a 13 μg/kg single IV bolus dose using 13-year-old, 50 kg male physiology.

Following successful preclinical plasma-model development and human MTM verification, the human PBPK model of MTMSA-Trp was used to project plasma and exploratory liver concentration-time profiles at 13 μg/kg IV bolus in a 13-year-old, 50 kg male using all three clearance prediction methods (Fig. 3, Fig. S4). Across all three methods, the model consistently projected substantially higher plasma exposure for MTMSA-Trp than for MTM at the same dose (Fig. 3, Table 3). Using the additional clearance approach, MTMSA-Trp achieved a 15-fold higher plasma exposure than MTM. Allometric scaling and single-species scaling yielded comparable MTMSA-Trp plasma exposures, approximately 8-fold higher than MTM. Model-derived liver exposures were 2- to 4-fold higher than MTM, and the predicted liver partition was lower for MTMSA-Trp (1% versus 6%); however, these tissue outputs should be interpreted cautiously because they are conditional on the assumed BPR and other distribution parameters. Taken together, all three clearance methods projected greater human plasma exposure of MTMSA-Trp relative to MTM at the same dose.

### 3.5. Parameter sensitivity analysis

The PSA of the human MTMSA-Trp PBPK model examined the influence of BPR, fup, and logP on predicted human plasma exposure (Fig. S5). Among these parameters, BPR was the most influential on the projected human PK profiles (Fig. S5a). Small changes in BPR produced large changes in the predicted concentration-time profile. In contrast, fup and logP showed comparatively limited sensitivity within the physiologically plausible ranges examined (Fig. S5b, S5c). Because the PSA was performed under one representative human-clearance scenario, these findings should be interpreted qualitatively.

## 4. Discussion

In this study, we developed and verified PBPK models for MTM and its semisynthetic analog MTMSA-Trp across preclinical species and then used the same workflow to project human plasma PK of MTMSA-Trp. The middle-out PBPK strategy was calibrated in mice, evaluated in rats and monkeys, and externally checked against clinical MTM plasma PK. Observed systemic clearance was used empirically within the model, while SpecPStc was optimized to mouse IV plasma PK and then applied across species. A permeability-limited tissue distribution model with Poulin and Theil tissue-to-plasma partition coefficient prediction was used because both compounds are relatively large, highly protein-bound molecules [12,18]. The substantially lower optimized SpecPStc for MTMSA-Trp is mechanistically consistent with its larger molecular weight and higher protein binding, both of which would be expected to reduce membrane permeability.

Based on their physicochemical properties, both compounds appear to belong to Class 3B of the Extended Clearance Classification System (ECCS), suggesting elimination driven predominantly by renal clearance, hepatic uptake, or a combination of both [19]. Mechanistic information on the specific clearance pathways of this class is limited; accordingly, the present model was designed as an empirically calibrated translational framework rather than a fully mechanistic elimination model. In the preclinical PBPK models, observed systemic clearance was used as an empirical input within the clearance term. For human MTM clearance prediction, the empirical allometric approaches failed to capture the observed clinical plasma PK. Given the marked interspecies differences in MTM clearance, this lack of allometric agreement was not unexpected. A renal-filtration-based clearance term also underpredicted MTM clearance across species, and an additional clearance term was therefore incorporated to capture the observed PK. Although this additional term was implemented mathematically alongside the renal clearance construct, it likely represents unidentified systemic elimination processes such as active renal secretion, hepatic uptake, or both. Incorporating this term using the average fractional contribution observed across preclinical species adequately captured human MTM plasma PK. Visual inspection, however, still suggested some underprediction of trough concentrations, indicating that aspects of distribution kinetics may remain incompletely captured.

Applying this workflow, the PBPK model projected substantially higher human plasma exposure for MTMSA-Trp than for MTM at the same dose. This projection was driven by lower predicted systemic clearance of MTMSA-Trp across all three clearance methods, as well as by higher lipophilicity and plasma protein binding. Higher protein binding reduces the free fraction available for renal filtration and other clearance mechanisms, resulting in lower systemic clearance and a longer plasma half-life. Because MTM exhibited high plasma clearance in humans and dose-limiting toxicity in the clinical study [11], the projection of materially higher MTMSA-Trp plasma exposure is translationally important. However, the current comparisons are based on total plasma and tissue concentrations. Given the markedly higher plasma protein binding of MTMSA-Trp, unbound systemic and tissue exposure may modify the translational interpretation and should be examined in future work. Similarly, the model-derived liver exposures and lower predicted liver partition for MTMSA-Trp are best viewed as exploratory and directionally informative rather than as validated evidence of a wider therapeutic window or reduced hepatotoxic risk.

In the monkey PBPK model, BPR for MTMSA-Trp had to be adjusted to a very low value to capture the observed plasma PK. This adjustment is mechanistically plausible because the fraction of MTMSA-Trp unbound is extremely low, favoring persistence in the plasma fraction rather than distribution into red blood cells. However, the need for this adjustment also highlights uncertainty in the model s distribution component. Because human BPR for MTMSA-Trp was not experimentally measured, human tissue exposure predictions remain conditional on this assumption. The PSA identified BPR as the most influential parameter among those examined, but that analysis was performed under a representative human-clearance scenario and should therefore be interpreted qualitatively. Additional uncertainty arises from the use of default GastroPlus^®^ physiological parameters and from the model s optimization primarily against plasma PK rather than tissue partition data. Prior preclinical modeling also suggested dose-dependent disposition of MTMSA-Trp in mice, with higher clearance observed at lower doses [4]; accordingly, the present human simulations should be interpreted as initial translational exposure projections at the dose range examined here rather than as definitive regimen-optimization results.

In conclusion, the successful development of a middle-out PBPK modeling strategy provides an empirically calibrated framework for translational evaluation of MTMSA-Trp in Ewing sarcoma. Following verification of the workflow against clinical MTM plasma PK, the MTMSA-Trp PBPK model projected substantially higher plasma exposure than MTM in humans. These results support the possibility of a favorable systemic exposure profile for MTMSA-Trp relative to MTM, while tissue exposure predictions, including liver partitioning, should be interpreted cautiously pending further characterization of compound-specific distribution behavior. As such, this model is best viewed as a tool to inform translational planning for first-in-human development.

## Supplementary Material

Supplementary Files

This is a list of supplementary files associated with this preprint. Click to download.

- NilloyetalSuppMtrlsTrpPBPK1.docx

## Figures and Tables

**Table 1.** Input parameters and source or status annotations for MTM and MTMSA-Trp PBPK models.

Parameter	MTM	MTMSA-Trp	Source / status
Molecular weight	1085.2	1227.3	-
logP	1.281	2.605	ADMET Predictor v11
Solubility (mg/mL)	0.965 at pH 5.359	0.416 at pH 5.721	ADMET Predictor v11
pKa	10.79 4.89	13.45, 10.81 6.79	ADMET Predictor v11 Measured
Mouse specific PStc (mL/s/mL)	2.95 × 10^−2^	1.23 × 10^−4^	Optimized to mouse plasma PK (objective function weight 1/Ŷ^2^, GastroPlus^®^ user manual)
Mouse fup (%)	2.55	<1 (assumed 0.1)	Measured [3]
Rat fup (%)	16.65	3.38	Measured [3]
Monkey fup (%)	11.86	1.06	Measured [3]
Human fup (%)	18.26	<1 (assumed 0.1)	Measured [3]
Mouse BPR	0.626	0.599	ADMET Predictor v11
Rat BPR	0.895	0.936	ADMET Predictor v11
Monkey BPR	0.671*	0.02^#^	*Assumed same as human; ^#^Adjusted to fit monkey plasma PK
Human BPR	0.671	0.661	ADMET Predictor v11
Kp prediction method	Poulin & Theil - extracellular	Poulin & Theil - extracellular	Predicted Kp method; permeability-limited model; Fu extracellular method = S+v9.5 (GastroPlus^®^ user manual)

**Table 2.** Prediction performance of the PBPK model for plasma PK.

Compound	Species (Dose)	AUC			Concentration	
		AUC_Obs_ (ng/mL.h)	AUC_Pred_ (ng/mL.h)	AUC_Pred/Obs_ Ratio	AFE	RMSE
MTM	Mouse (2 mg/kg)	1906	3222	1.69	1.84	0.36
	Rat (0.5 mg/kg)	173	234	1.35	1.39	0.86
	Monkey (6.94 μg/kg)	90	147	1.63	1.62	0.26
	Monkey (13.18 μg/kg)	255	278	1.09	1.30	0.34
	Human (13 μg/kg)*	136	143	1.05	0.52	0.61
MTMSA-Trp	Mouse (2 mg/kg)	38563	50807	1.32	1.48	0.26
	Rat (0.5 mg/kg)	3486	3569	1.02	0.85	0.33
	Monkey (1.34 μg/kg)	139	65	0.46	0.73	0.33
	Monkey (4.19 μg/kg)	181	249	1.38	1.46	0.25

* AUCs were calculated using extracted or predicted concentrations for 24 hours following the first dose. AFE and RMSE were calculated using extracted or predicted concentration-time points across the 7-day dosing period. Mouse values reflect calibration for SpecPStc optimization, whereas rat and monkey values reflect cross-species evaluation; human MTM values reflect clinical plasma PK verification.

**Table 3.** Model-derived exposure comparison based on human PBPK simulation.

Compound	Clearance Prediction method	AUC_Plasma_	AUC_Liver_	Liver partition	AUC_Plasma_/AUC_Plasma(MTM)_	AUC_Liver_/AUC_Liver(MTM)_
MTM	Additional clearance	170	10	0.06	1	1
MTMSA-Trp	Additional clearance	2604	39	0.01	15.3	3.9
	Allometric scaling	1325	20	0.01	7.8	2
	Single species scaling	1365	20	0.01	8	2

AUCs were estimated over 24 hours; liver partition = AUC_Liver_ / AUC_Plasma_. Liver exposure outputs are model-derived and exploratory.

## Data Availability

Inquiries regarding the datasets should be directed to the corresponding author.
